# Two new species of the genus *Symphylella* (Symphyla, Scolopendrellidae) from Tibet, China

**DOI:** 10.3897/zookeys.845.33566

**Published:** 2019-05-15

**Authors:** Ya-Li Jin, Yun Bu, Yue Jiang

**Affiliations:** 1 Natural History Research Center, Shanghai Natural History Museum, Shanghai Science & Technology Museum, Shanghai, 200041, China Shanghai Natural History Museum, Shanghai Science & Technology Museum Shanghai China; 2 Shanghai Investigation, Design & Research Institute Co., Ltd., Shanghai 200434, China Shenzhen Techand Ecology & Environment Co. LTD Shenzhen China

**Keywords:** Chaetotaxy, key, Myriapoda, sensory organ, taxonomy, Tömösváry organ

## Abstract

The Symphyla of Tibet are studied for the first time. *Symphylellamacropora***sp. n.** and *Symphylellazhongi***sp. n.** from southeastern Tibet are described and illustrated. *Symphylellamacropora***sp. n.** is characterized by large, elongated oval openings of the Tömösváry organ with its inner margins covered by minute irregular teeth, rudimentary spined sensory organs present on the dorsal side of most antennal segments, and cerci with numerous long and slightly curved setae. *Symphylellazhongi***sp. n.** is characterized by a globular Tömösváry organ with a small and roundish opening, mushroom-shaped sensory organs present on apical antennal segments, and by having tergal processes longer than their basal width with ovoid swollen ends. The newly described species are compared to the morphologically closest congeners: *S.javanensis*, *S.asiatica*, *S.multisetosa*, and *S.simplex*. A key for 43 species of the genus is also provided.

## Introduction

Knowledge of the myriapod class Symphyla from China is poor. Only two species have been recorded until now: *Hanseniellacaldaria* (Hansen, 1903) from Zhejiang province and *Scolopendrellopsisglabrus* Jin & Bu, 2018 from Zhejiang and Hainan provinces (Zhang and Wang 1992; Bu and Jin 2018; Jin and Bu 2018). There are no records of Symphyla from Tibet so far.

During an investigation of soil arthropods in southeast Tibet in November 2015, plenty of specimens of the family Scolopendrellidae were obtained. Among them, two new species of the genus *Symphylella* Silvestri, 1902 were identified and are described here.

## Materials and methods

Specimens were collected by Berlese-Tullgren funnels and preserved in 80% ethanol. They were mounted under slides using Hoyer’s solution and dried in an oven at 60 °C. Observations were performed under a phase-contrast microscope (Leica DM 2500). Photographs were taken by a digital camera installed on the microscope (Leica DMC 4500). Line drawings were done using a drawing tube. All specimens are deposited in the collections of Shanghai Natural History Museum (SNHM), Shanghai, China.

Abbreviations used in this paper: ***al***-anterolateral seta, ***as***-apical seta, ***bo***-bladder-shaped organ, ***co***-cavity-shaped organ, ***cs***-central seta, ***ibs***-inner basal seta, **is**-inserted seta, ***lms***-lateromarginal seta, ***mo***-mushroom-shaped organ, ***rso***-rudimentary spined sensory organ, ***so***-spined sensory organ.

## Results

### Taxonomy

#### Family Scolopendrellidae Bagnall, 1913

##### Genus Symphylella

Taxon classificationAnimaliaSymphylaScolopendrellidae

Silvestri, 1902

Type species: Symphylellaisabella (Grassi, 1886)

###### Diagnosis.

Central rod on head broken and distinct in both anterior and posterior portions. Antennae with 14–22 segments. Trunk with 17 tergites or fewer, with the first tergite vestigial. Triangular processes present on posterior margins of 13 tergites. Belts of longitudinal striae between processes absent. First pair of legs vestigial, as small protuberances with a few setae. Styli rudimentary. Coxal plates with sacs only present on 3^rd^–9^th^ legs. Cerci relatively long, terminal area with transverse stripes, ending in a single long seta (Bagnall 1913; Szucsich and Scheller 2011).

###### Distribution.

The genus *Symphylella* currently includes 47 extant subcosmopolitan species (Szucsich and Scheller 2011; Jin and Bu 2018). It has previously been recorded from China, but the species have never been identified (Zhang and Wang 1992).

##### Symphylella macropora

Taxon classificationAnimaliaSymphylaScolopendrellidae

Jin & Bu sp. n.

http://zoobank.org/7AAA91E2-37AF-45CE-B14F-9433EBD3FF75

**Figure 1.**
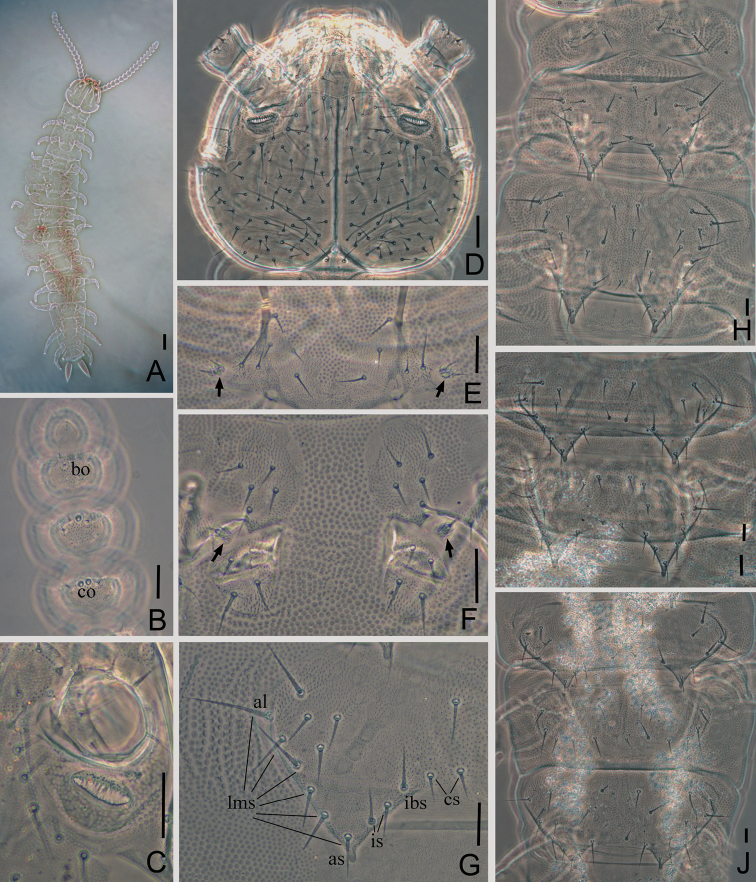
*Symphylellamacropora* sp. n. **A** habitus **B** left antenna, 15^th^–18^th^ segment, dorsal view (*bo*-bladder-shaped organ, *co*-cavity-shaped organ) **C** right Tömösváry organ **D** head, dorsal view **E** first pair of legs (arrows indicate the legs) **F** stylus and coxal sacs of leg 3 (arrows indicate styli) **G** 4^th^ tergite, left side (*al*-anterolateral seta, *as*-apical seta, *cs*-central seta, *ibs*-inner basal seta, *is*-inserted seta, *lms*-lateromarginal seta) **H** 1^st^–3^rd^ tergites **I** 4^th^–5^th^ tergites **J** 13^th^–15^th^ tergites. Scale bars: 100 μm (**A**); 20 μm (**B–J**).

**Figure 2.**
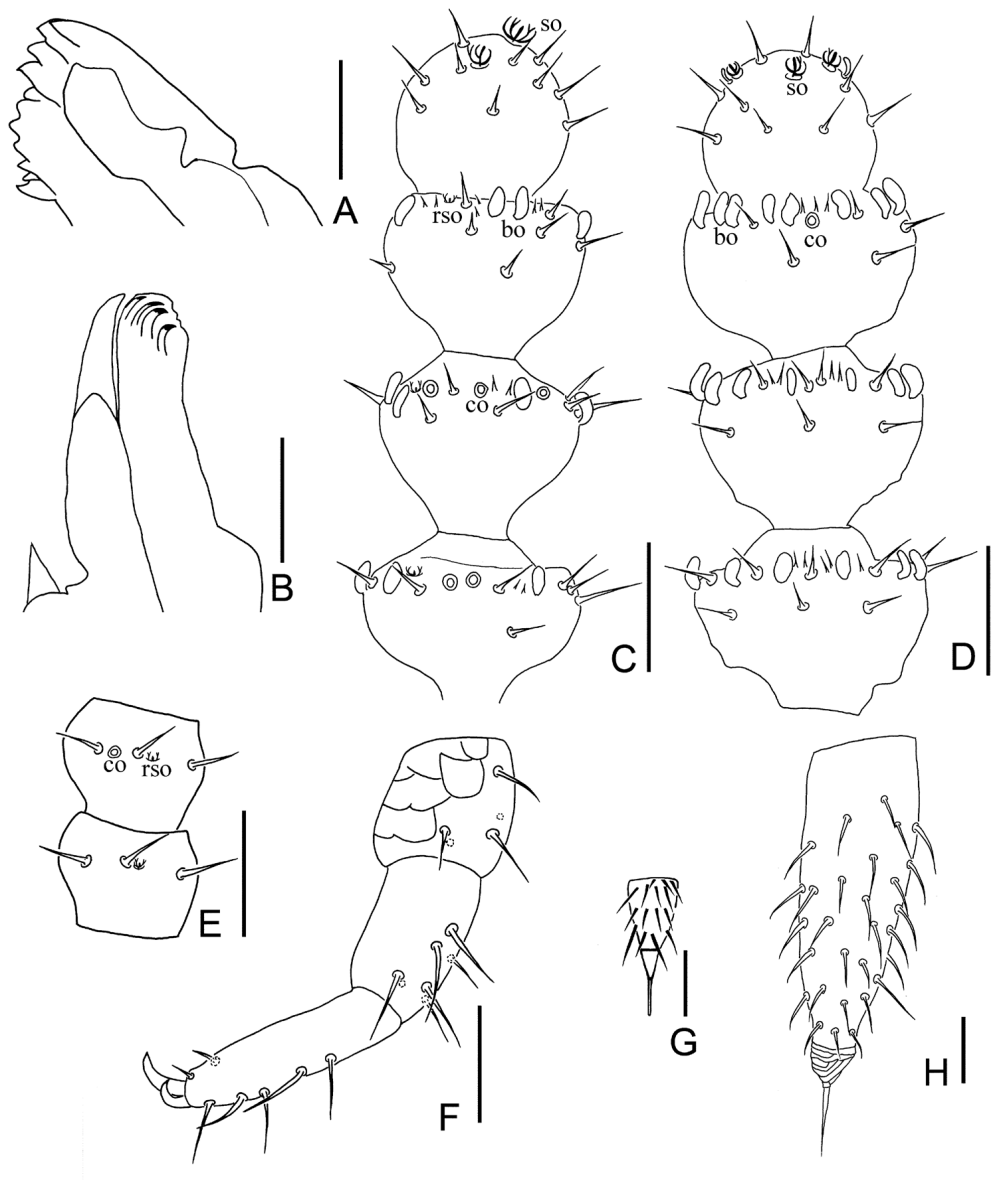
*Symphylellamacropora* sp. n. **A** mandible **B** first maxilla **C–D** 15^th^–18^th^ segments of left antenna **C** dorsal view (*bo*-bladder-shaped organ, *co*-cavity-shaped organ, *rso*-rudimentary spined sensory organ, *so*-spined sensory organ) **D** ventral view **E** 4^th^–5^th^ segments of right antenna, dorsal view **F** 12^th^ leg **G** stylus on base of 4^th^ leg **H** right cercus, dorsal view. Scale bars: 20 μm (**A–F, H**); 5 μm (**G**).

Figures 1 , 2 , Tables 1 , 2 , 3

###### Diagnosis.

*Symphylellamacropora* sp. n. is characterized by large, elongated oval openings of the Tömösváry organs, with their inner margins of these openings covered by minute irregular teeth, as well as by having rudimentary spined sensory organs on the dorsal side of most antennal segments, and cerci with numerous long and slightly curved setae.

###### Material examined.

Holotype, female (slide no. XZ-SY2015030) (SNHM), China, Tibet, Motuo county, Dexing town, extracted from soil samples from broadleaf forest, alt. 1100 m, 29°40'N, 95°26'E, 3-XI-2015, coll. Y. Bu & G. Yang. Paratypes, 6 females (slides nos. XZ-SY2015023–XZ-SY2015026, XZ-SY2015029, XZ-SY2015032) (SNHM), 3 males (slides nos. XZ-SY2015027, XZ-SY2015028, XZ-SY2015031) (SNHM), same date as holotype; 1 female (slide no. XZ-SY2015034) (SNHM), China, Tibet, Motuo county, Beibeng town, extracted from soil samples from broadleaf forest, alt. 1500 m, 29°30'N, 95°38'E, 5-XI-2015, coll. Y. Bu & G. Yang. Other materials (SNHM): 1 juvenile with 9 pairs of legs (slide no. XZ-SY2015033) (SNHM), China, Tibet, Motuo county, Beibeng town, extracted from soil samples from broadleaf forest, alt.1500 m, 29°30'N, 95°38'E, 5-XI-2015, coll. Y. Bu & G. Yang; 2 juveniles with 9–10 pairs of legs (slides nos. XZ-SY2015035–XZ-SY2015036) (SNHM), same date as holotype; 10 juveniles with 8–10 pairs of legs (slides nos. XZ-SY2015037–XZ-SY2015046) (SNHM), China, Tibet, Motuo county, Dexing town, Naerdong village, extracted from soil samples from broadleaf forest, alt. 1800 m, 29°30'N, 95°23'E, 4-XI-2015, coll. Y. Bu.

###### Description.

Adult body 1.90 mm long in average (1.55–2.71 mm, n=11), holotype 1.89 mm (Fig. 1A).

*Head* length 210–270 μm, width 225–300 μm, with widest part somewhat behind the middle on a level with the points of articulation of mandibles. Central rod distinct in both anterior (50–70 μm) and posterior (58–75 μm) portions, with a middle knot. Anterior branches well developed, median branches vestigial. Head dorsally moderately covered with setae of different length, longest setae (25–35 μm) located most anteriorly, at least 3.0 times as long as central ones (8–12 μm). Cuticle at anterolateral part of head with rather coarse granulation. Tömösváry organ surrounded by fine granulation, other areas with fine dense granulation (Fig. 1D).

*Tömösváry organ* oval, length 19–32 μm, width 10–22 μm, at least half of greatest diameter of 3^rd^ antennal segment (35–50 μm), openings large and elongated oval (length 13–26 μm, width 5–10 μm), with their inner margins covered by minute irregular teeth (Figs 1C, 1D).

*Mouthparts* Mandible with two fused lamellae and 11 teeth in total (Fig. 2A). First maxilla with two lobes, inner lobe with four hook-shaped teeth, palp bud-like with 1 distal point close to outer lobe (Fig. 2B). Anterior part of second maxilla with many small protuberances which carry one seta each, distal setae thickened; posterior part with sparse setae. Cuticle of maxilla and labium covered with pubescence.

*Antennae* with 16–23 segments (18 in holotype), length 570–680 μm (620 μm in holotype), about 0.3 of body length. 1^st^ segment cylindrical, much shorter than following ones, greatest diameter wider than long (37–45 μm, 13–33 μm), with 2–3 microsetae and 6–9 long mesosetae in one whorl, longest seta (20–24 μm) inserted at inner side and distinctly longer than outer ones (15–18 μm). 2^nd^ segment wider (35–44 μm) than long (28–35 μm), with eight or nine setae evenly inserted around antennal wall, interior setae (23–26 μm) slightly longer than exterior ones (16–19 μm). Chaetotaxy of 3^rd^ segment like preceding ones. Setae on basal segments slender and on distal segments shorter. Basal segments of antennae with only primary whorl of setae, in middle and subapical segments with several minute setae in secondary whorl. Three kinds of sensory organs on most antenna segments: *rso* on dorsal side of most segments (Figs 2C, 2E); *co* on dorsal side of 3^rd^–21^th^ segments; *bo* on subapical 5–9 segments (Figs 1B, 2C, 2D). Apical segment subspherical, somewhat wider than long (width 28–32 μm, length 24–26 μm), with 13–15 setae on distal half and 2 baculiform organs on apex, 4–7 *so* consisting of 3–4 curved spines around a central pillar in depressions in distal surface (Figs 2C, 2D). All segments covered with short pubescence. Chaetotaxy and sensory organs of antennae are given in table 1.

**Table 1.** Numbers of setae and sensory organs of antennae (holotype).

**Segments**	**Nos. of primary whorl setae**	**Nos. of secondary whorl setae**	**Rudimentary spined sensory organs (rso)**	**Cavity-shaped organs (co)**	**Bladder-shaped organs (bo)**	
				**Dorsal**	**Dorsal**	**Ventral**
1^st^	6		1			
2^nd^	8		1			
3^rd^	8		1			
4^th^	9		1			
5^th^	10		1			
6^th^	10		1			
7^th^	11		1			
8^th^	11		1			
9^th^	11		1			
10^th^	11		1			
11^th^	11	3	1			
12^th^	12	4	1			
13^th^	12	4	1	2	2	
14^th^	12	4	1	3	3	
15^th^	12	4	1	9	3	6
16^th^	12	4	1	11	4	7
17^th^	12	5	1		4	8

*Trunk* with 17 tergites. Most tergites overlap, with intertergal zones present between adjacent tergites, except for borders between 14^th^ and 15^th^, as well as 16^th^ and 17^th^ tergite. Tergites 2–13, and 15 each with one pair of triangular processes. Basal distance between processes of 4^th^–13^th^and 15^th^ tergites longer than their length from base to tip, and the latter shorter than its basal wide (Table 2). Anterolateral setae of 2^nd^, 3^rd^, 4^th^, 6^th^, 7^th^, 9^th^ and10^th^ tergites distinctly longer than other lateromarginal setae, that of 5^th^, 8^th^, 11^th^–13^th^ and 15^th^ subequal or slightly longer than other lateromarginal setae. Triangular processes without ovoid swollen ends (Fig. 1G). One to three inserted setae (setae between inner basal seta and apical seta). All tergites pubescent.

**Table 2.** Chaetotaxy of tergites (holotype in brackets).

**No. of tergites**	**Lateromarginal setae**	**Inserted seta**	**Central setae**	**Other setae**
1^st^				
2^nd^	5–8 (5)	1–2 (1)	1–4 (1)	6–13 (8)
3^rd^	6–10 (6–8)	1–3 (1)	1–4 (1)	14–25 (14)
4^th^	5–7 (5)	1–3 (1)	2–5 (2)	7–15 (7)
5^th^	5–8 (5)	1–4 (1)	2–5 (2)	9–15 (9)
6^th^	8–11 (8)	1–3 (1)	2–6 (2)	16–36 (16)
7^th^	4–8 (4)	1–3 (1)	4–7 (4)	9–15 (9)
8^th^	5–9 (5–6)	1–3 (1)	3–5 (3)	8–17 (8)
9^th^	8–12 (8)	1–3 (1)	3–6 (3)	14–32 (14)
10^th^	5–7 (6)	1–2 (1)	3–6 (3)	7–15 (7)
11^th^	5–8 (5)	1–3 (1)	2–6 (2)	7–16 (7)
12^th^	6–10 (6–7)	1–3 (1)	2–6 (2)	16–31 (16)
13^th^	4–7 (4)	0–2 (0–1)	2–5 (2)	6–13 (6)
14^th^	11–17 (11)*			3–11 (3)
15^th^	5–9 (5–6)	0–2 (0–1)	1–3 (1)	11–24 (11)
16^th^	6–14 (6)*			2–7 (2)
17^th^				20–31(20)

Note: * – marginal setae.

*Tergites* 1^st^ tergite reduced and build up of two short plates separated in the median axis of the body, with 6–9 short setae in a row. 2^nd^ tergite complete, with two posterior processes, 5–8 lateromarginal setae, 1–2 inserted setae, 1–4 central setae, with anterolateral setae distinctly longer than other lateromarginal setae, processes approximately 0.9 times as long as broad, basal distance between processes approximately 0.7 times as long as their length. 3^rd^ tergite entire, broader and longer than preceding one with ratios mentioned nearly the same as for the 2^nd^ tergite, 6–10 lateromarginal setae (Fig. 1H). 4^th^ tergite broader than 3^rd^ tergite, with ratios approximately 0.7 and 1.4 respectively, 5–7 lateromarginal setae (Fig. 1I). Shape and chaetotaxy of 5^th^–7^th^, 8^th^–10^th^, and 11^th^–13^th^ tergite similar as 2^nd^–4^th^ tergite. 15^th^ tergite shorter with smaller processes than remaining long tergites (3^rd^, 6^th^, 9^th^ and 12^th^ tergites). 14^th^ and 16^th^ tergites without processes, with 11–17 and 6–14 marginal setae respectively (Fig. 1J). 17^th^ tergite with 20–31 setae. Chaetotaxy and measurements of tergites are given in Table 2, 3.

**Table 3.** Measurements of tergites and processes (mean ± se, *n* = 11) (in μm).

**No. of tergites**	**Length**	**Width**	**Length of processes**	**Basal width of processes**	**Basal distance between processes**
1^st^	35.6±3.1	148±9.9			
2^nd^	62.9±4.6	146.7±4.7	39.5±1.9	45.4±2.8	27.6±1.4
3^rd^	107.5±6.2	179.3±4.9	42±1.7	50.8±2.7	30.8±1.6
4^th^	71.2±7.6	193.8±10.2	37±1.5	55±2.9	53.1±1.5
5^th^	80±6.8	189±5.9	42±0.8	52.6±2.5	53.7±2.9
6^th^	122.9±9.3	229.3±8.4	45.7±1.3	54.3±2	58.9±2.4
7^th^	78.6±8	237.3±8.1	38.6±0.8	60.5±3.6	71.3±2.2
8^th^	90.1±8	209.5±6.8	40.9±1.5	51.7±2.4	65.7±3.7
9^th^	138.1±9.5	245.3±8.6	45.7±1.7	56.6±2.6	64.7±3.7
10^th^	76.4±9	252.6±9.7	37.6±1.3	58.7±3.2	80.5±3.1
11^th^	85.7±6.9	204.7±10.5	42.6±1.6	55.8±2.8	71.4±2.5
12^th^	121.7±7.7	258.7±7.6	41.5±2.1	60±4.1	70.3±3.2
13^th^	75±6	242.9±8.9	32.5±1.9	57.9±3.4	75.9±3.6
14^th^	73.1±5.4	202.7±5.8			
15^th^	88.7±7.1	210.2±13.7	32±1.9	49.3±3.3	56.4±3.9
16^th^	62.2±2.7	170.7±11.6			
17^th^	104.8±2.7	143.8±10.3			

*Legs* 1^st^ pair of legs reduced to two small hairy cupules, each with two long setae (Fig. 1E). Basal areas of legs 2–12 each with 4–6 setae (Fig. 1F). Leg 12 about 0.1 time as long as body, subequal length with head. Trochanter longer than wide (52–76 μm, 40–56 μm) with 6–8 subequal setae. Femur as long as wide (32–42 μm, 30–41 μm), with 4–6 setae and one of them (17–28 μm) distinctly longer than others (10–20 μm); pubescent dorsally, ventrally with cuticular thickenings in pattern of large scales. Tibia nearly 2 times longer than wide (40–60 μm, 23–30 μm), with 5–7 dorsal setae and the longest one slightly shorter than greatest diameter of tibia (19–28 μm).Tarsus subcylindrical, at least 3 times as long as wide (50–70 μm, 15–20 μm), with 6–9 setae: 3 straight and protruding, 2 curved and depressed; longest setae (20–27 μm) about same length of greatest width of podomere, and 2 ventral setae inserted near claw distinctly shorter than dorsal ones. Claws rather curved, anterior one distinctly longer and broader than posterior one, the latter more curved than the former (Fig. 2F). All legs covered with dense pubescence except areas with cuticular thickenings.

*Coxal sacs* present at bases of 3^rd^–9^th^ pairs of legs, fully developed, each with 4 setae on its surface (Fig. 1F).

*Styli* present at base of 3^rd^–12^th^ pairs of legs, subconical (length 6–9 μm, width 4–6 μm), basal part with straight hairs; distal one fourth hairless and blunt (3–6 μm) (Figs 1F, 2G).

*Sensecalicles* with smooth margin to pit, length about the same as outer diameter (18–39 μm, 20–35 μm). Sensory seta inserted in cup center, extremely long, length 130–165 μm, at least 5.5 times longer than other two lateral setae (20–35 μm, 14–22 μm respectively) that inserted at the edge of cup.

*Cerci* subuliform, short, about 0.6 of head length and leg 12, length at least three times as long as its greatest width (126–172 μm, 40–53 μm), moderately covered with subequal length and slightly curved setae, with longest (20–40 μm) at least half of the greatest width of the cerci, terminal area (23–32 μm) short, circled by 6–8 layers of curved ridges. Terminal setae length 18–28 μm (Fig. 2H).

###### Etymology.

The species name *macropora* is referring to the large opening of the Tömösváry organ.

###### Distribution.

Known only from the type locality.

###### Remarks.

*Symphylellamacropora* sp. n. is most similar to *S.javanensis* Scheller, 1988 from Java in the shape of tergites and processes, leg 12 and sensory organs on antennae. However, it can be readily distinguished from the latter by the shape of Tömösváry organ (oval, openings large and elongated with inner margins covered by minute irregular teeth vs subspherical, diameter of opening about half of the organ in *S.javanensis*), central rod (both anterior and posterior portions distinct in *S.macropora* sp. n. vs anterior half and anterior branches very thin with traces in *S.javanensis*), and the stylus (with blunt apex in *S.macropora* sp. n. vs with truncate apex in *S.javanensis*). It is also closely related to *S.asiatica* Scheller, 1971 from Indiaand Sri Lanka in the shape and chaetotaxy of tergites 1–3, leg 12 and the sensory organs on antenna, but easily distinguished by characters of Tömösváry organ (openings very small in *S.asiatica*), and the cerci (most setae subequal length and slightly curved in *S.macropora* sp. n. vs long and erect setae on dorsal, ventral and outer sides arranged in one or two rows in *S.asiatica*).

##### Symphylella zhongi

Taxon classificationAnimaliaSymphylaScolopendrellidae

Jin & Bu sp. n.

http://zoobank.org/159AD15C-EB70-409D-8E5F-3A1E25B62C22

**Figure 3.**
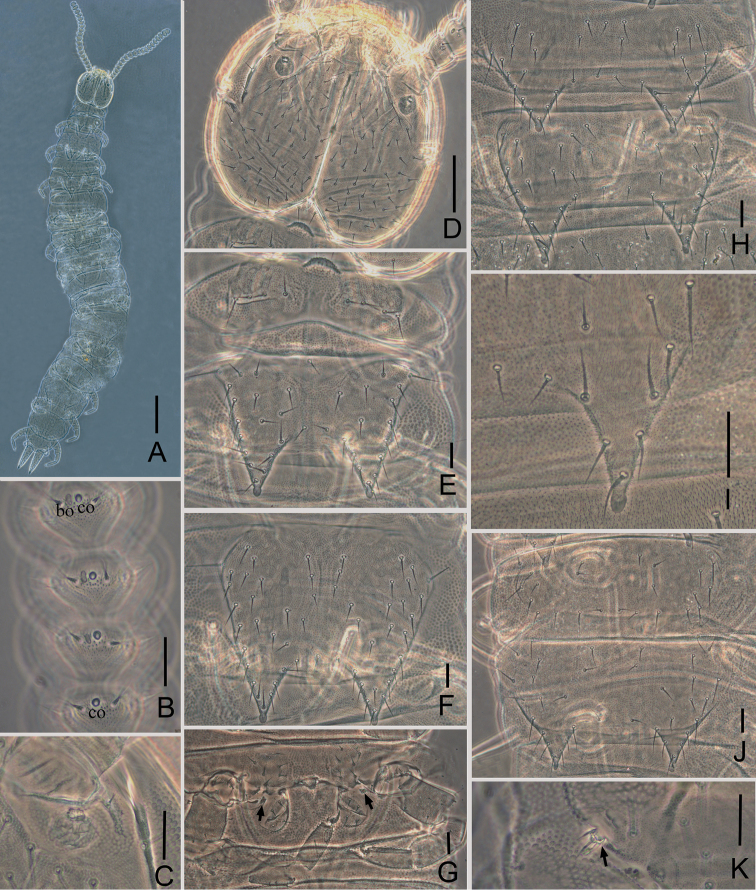
*Symphylellazhongi* sp. n. **A** habitus **B** right antenna, 8^th^–11^th^ segments, dorsal view (*bo*-bladder-shaped organ, *co*-cavity-shaped organ) **C** right Tömösváry organ **D** head, dorsal view **E** 1^st^–2^nd^ tergites **F** 3^rd^ tergite **G** female gonopore, styli, and coxal sacs on base of leg 4 (arrows indicate styli) **H** 4^th^–5^th^ tergite **I** 8^th^ tergite, right side **J** 14^th^–15^th^ tergites **K** leg 1 (arrow indicates the leg). Scale bars: 200 μm (**A**); 20 μm (**B–K**).

**Figure 4.**
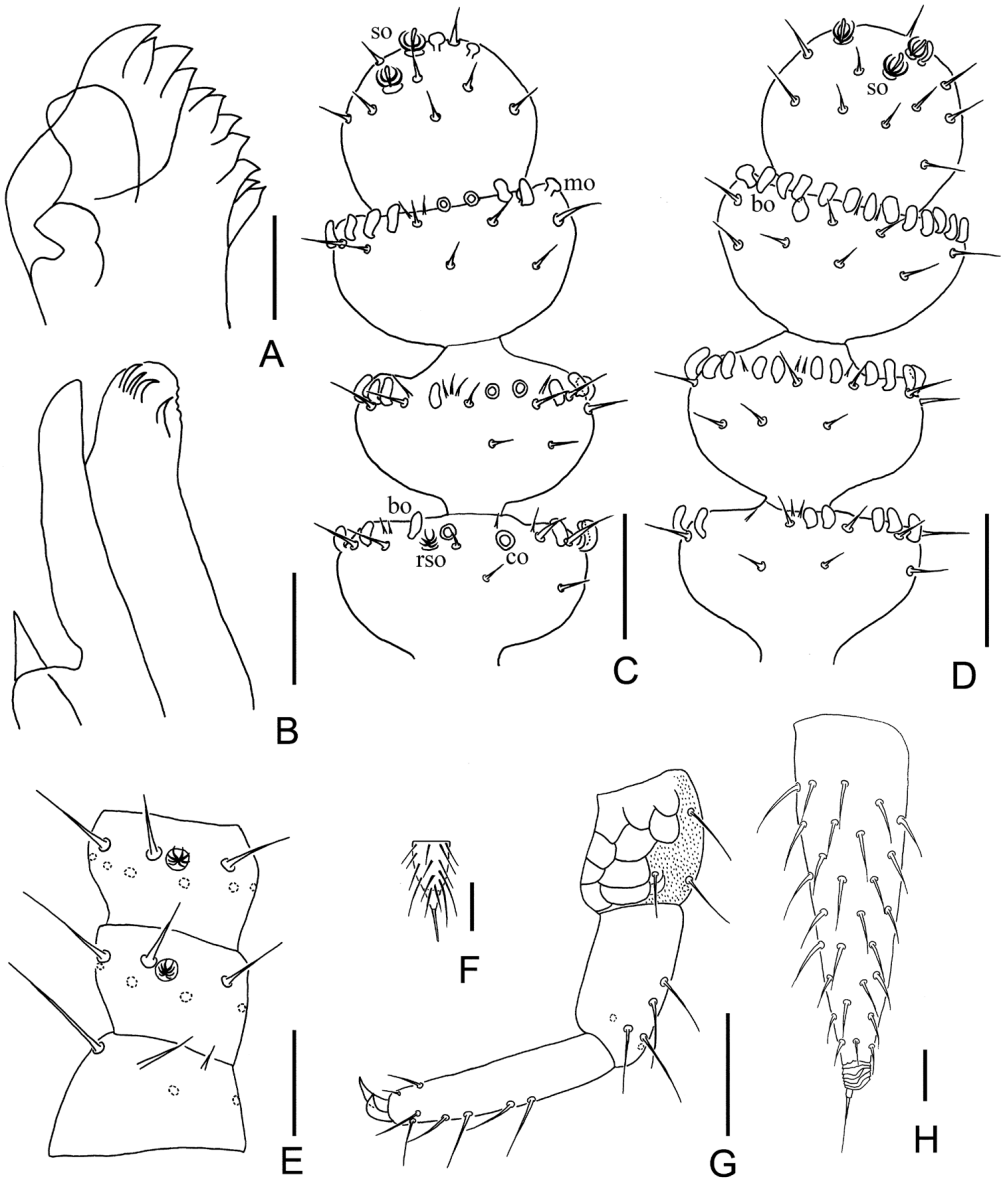
*Symphylellazhongi* sp. n. **A** mandible **B** first maxilla **C–D** 18^th^–21^th^ segments of right antenna **C** dorsal view (*bo*-bladder-shaped organ, *co*-cavity-shaped organ, *mo*-mushroom-shaped organ, *rso*-rudimentary spined sensory organ, *so*-spined sensory organ) **D** ventral view **E** 1^st^–3^rd^ segments of right antenna, dorsal view **F** stylus on base of 5^th^ leg **G** 12^th^ leg **H** right cercus, dorsal view. Scale bars: 20 μm (**A–E, G, H**); 5 μm (**F**).

Figures 3 , 4 , Tables 4 , 5 , 6 , 7

###### Diagnosis.

*Symphylellazhongi* sp. n. is characterized by a globular Tömösváry organ with small and roundish opening, processes on tergites mostly longer than their basal width, ovoid swollen ends of processes, and mushroom-shaped sensory organs present on apical antennal segments.

###### Material Examined.

Holotype, female (slide no. XZ-SY2015049) (SNHM), China, Tibet, Linzhi City, Bomi county, Songzong town, extracted from soil samples from broadleaf forest, alt. 3000 m, 29°76'N, 95°96'E, 7-XI-2015, coll. Y. Bu & G. Yang. Paratypes, 2 females (slides nos. XZ-SY2015047–XZ-SY2015048) (SNHM), same date as holotype. Other materials: 3 juvenile with 8 or 9 pairs of legs (slides nos. XZ-SY2015050–XZ-SY2015052) (SNHM), same date as holotype.

###### Description.

Adult body 2.48 mm long on average (2.22–2.93 mm, *n* = 3), holotype 2.93 mm (Fig. 3A).

*Head* length 250–262 μm, width 262–287 μm, with widest part somewhat behind the middle on a level with the points of articulation of mandibles. Central rod distinct in both anterior (65–70 μm) and posterior (75–90 μm) portions, with a middle knot. Anterior branches well developed, median branches vestigial. Dorsal side of head moderately covered with setae of different length, longest setae (27–35 μm) located most anterior on head, at least 3.0 times as long as central ones (8–11 μm). Cuticle at anterolateral part of head with rather coarse granulation. Tömösváry organ surrounded by fine granulation, other area with faint dense granulation (Fig. 3D).

*Tömösváry organ* globular, length19–24 μm, width 16–22 μm, about half of greatest diameter of 3^rd^ antennal segments (40–42 μm), opening small and roundish (length 8 μm, width 3–6 μm) (Figs 3C, D).

*Mouthparts.* Mandible with two fused lamellae and 11 teeth in total (Fig. 4A). First maxilla has two lobes, inner lobe with four hook-shaped teeth, palp bud-like with one distal point close to outer lobe (Fig. 4B). Anterior part of second maxilla with many small protuberances which carry one seta each, distal setae more thick and hard; posterior part with sparse setae. Cuticle of maxilla and labium covered with pubescence.

*Antennae* with 18–23 segments (left antenna with 19, right antenna with 21 in holotype), length 513–663 μm (663 μm in holotype), about one fourth of body length. 1^st^ segment cylindrical, greatest diameter somewhat wider than long (40–42 μm, 23–40 μm), with 1 or 2 microsetae and 6 or 7 mesosetae in one whorl, longest seta (20–22 μm) inserted at inner side and distinctly longer than outer ones (14–15 μm). 2^nd^ segment wider (25–28 μm) than long (40 μm), with 7 or 8 setae evenly inserted around the antennal wall with interior setae (20–21 μm) slightly longer than exterior ones (14–15 μm). Chaetotaxy of 3^rd^ segment similar to preceding ones. Setae on basal segments longer and on distal segments shorter. Basal antennae segments with only primary whorl of setae, in middle and subapical segments several setae in secondary whorl. Four kinds of sensory organs on antenna: *rso* on dorsal side of most segments (Figs 4C, E); *co* on 7^th^–19^th^ segments; *bo* on 9–11 segments next to apical one increasing in number on subdistal segments to a maximum of 21 (Figs 3B, 4C); *mo* on distal 2 segments (Fig. 4C). Apical segment subspherical, somewhat longer than wide (width 24–27 μm, length 32–35 μm), with 15–18 setae on distal half; 3–5 spined sensory organs consisting of 3 or 4 curved spines around a central pillar in depressions in distal surface; 2 baculiform organs on apex of segment (Figs 4C, D). All segment covered with short pubescence. Chaetotaxy and sensory organs of antennae are given in Table 4.

**Table 4.** Numbers of setae and sensory organs of antennae (holotype).

**Segments**	**No. of primary whorl setae**	**No. of secondary whorl setae**	**Rudimentary spined sensory organs (rso)**	**Cavity-shaped organs on dorsal side (co)**	**Bladder-shaped organs (bo)**		**Mushroom-shaped organs (mo)**
					**Dorsal**	**Ventral**	
1^st^	7						
2^nd^	8		1				
3^rd^	8		1				
4^th^	9		1				
5^th^	9		1				
6^th^	11		1				
7^th^	11		1	1			
8^th^	11			1			
9^th^	11	1		1	1		
10^th^	11	1	1	1	1		
11^th^	12	4	1	1	2	1	
12^th^	12	4	1	1	2	2	
13^th^	10	5	1	1	2	1	
14^th^	10	5	1	1	3	2	
15^th^	10	5	1	1	3	3	
16^th^	10	5	1	1	5	5	
17^th^	12	5	1	2	5	12	
18^th^	12	5		2	9	14	
19^th^	12			2	7	14	2
20^th^	12						2

*Trunk* with 17 dorsal tergites. Most tergites overlap, with intertergal zones present between adjacent tergites, except for borders between 14^th^ and 15^th^, as well as 16^th^ and 17^th^ tergite. Tergites 2–13 and 15 each with one pair of triangular processes. Length from base to tip of processes somewhat longer than its basal width except for the 4^th^, 7^th^, 10^th^ and 13^th^ tergites, where processes are broader than long; basal distance between processes of 4^th^–13^th^ and 15^th^ tergite longer than their length from base to tip (Table 5). Triangular processes with ovoid swollen ends (Fig. 3I). Anterolateral setae of 2^nd^, 3^rd^, 4^th^, 6^th^, 7^th^, 9^th^ and10^th^ tergite distinctly longer than other lateromarginal setae, that of 5^th^, 8^th^, 11^th^ –13^th^ and 15^th^ subequal or slightly longer than other lateromarginal. One to two inserted setae. All tergites pubescent.

**Table 5.** Measurements of tergites and processes (in μm, *n* = 3) (holotype in brackets).

**No. of tergites**	**Length**	**Width**	**Length of processes**	**Basal width of processes**	**Basal distance between processes**
1^st^	35–50 (50)	160–175 (160)			
2^nd^	75–80 (75)	160–180 (180)	43–50 (50)	38–45 (45)	38–40 (40)
3^rd^	75–145 (145)	190–230 (206)	53–58 (55)	43–50 (50)	40–50 (50)
4^th^	75–96 (96)	220–236 (236)	45–50 (50)	48–58 (58)	70–75 (75)
5^th^	76–95 (95)	200–232 (232)	50–60 (60)	45–53 (53)	60–78 (78)
6^th^	125–156 (156)	283–310 (310)	60–60 (60)	53–58 (58)	55–88 (88)
7^th^	75–90 (90)	270–300 (300)	50–53 (53)	53–60 (60)	73–100 (100)
8^th^	90–100 (95)	246–264 (264)	53–60 (55)	53–55 (53)	73–105 (105)
9^th^	158–160 (158)	310–370 (370)	60–63 (63)	50–58 (58)	88–100 (100)
10^th^	97–114 (114)	300–350 (350)	48–58 (58)	55–63 (63)	100–110 (110)
11^th^	88–100 (100)	246–272 (272)	53–63 (63)	48–60 (60)	88–110 (110)
12^th^	150–190 (190)	312–334 (312)	53–60 (58)	50–55 (50)	83–110 (110)
13^th^	85–150 (150)	280–330 (330)	48–50 (50)	53–63 (63)	88–113 (113)
14^th^	101–142 (142)	220–276 (276)			
15^th^	110–190 (190)	260–328 (328)	45–60 (45)	45–58 (45)	73–95 (95)
16^th^	78–135 (135)	220–280 (280)			
17^th^	140–170 (170)	180–195 (195)			

*Tergites.* 1^st^ tergite reduced and build up of two short plates separated in the median axis of the body, with 8 short setae in a row. 2^nd^ tergite complete, with two slender posterior processes, 6–8 lateromarginal setae, 1 or 2 inserted setae, 2 central setae, with anterolateral setae distinctly longer than other lateromarginal ones, processes approximately 1.1 times as long as broad, basal distance between processes approximately 0.8 times as long as their length (Fig. 3E). 3^rd^ tergite complete, broader and longer than preceding one with ratios of 1.1 and 0.9 respectively, 8 or 9 lateromarginal setae (Fig. 3F). 4^th^ tergite broader than 3^rd^ tergite, with ratios approximately 0.8 and 1.5 respectively, 6 or 7 lateromarginal setae (Fig. 3H). Chaetotaxy of 5^th^–7^th^, 8^th^–10^th^, and 11^th^–13^th^ tergites similar to 2^nd^–4^th^ tergites. Pattern of alternating tergite lengths of two short tergites followed by a long tergite only disrupted at the caudal end (Table 5). Within short tergites (1, 2, 4, 5, 7, 8, 10, 11, 13, 14, 16) the length augments toward caudal. Same is generally true for long tergites (3, 6, 9, 12, 15, 17), but for the 15^th^ tergite being shorter than the others, with likewise smaller processes. 14^th^ and 16^th^ tergite without processes with 13–18 and 10–15 marginal setae respectively (Fig. 3J). 17^th^ tergite with 32–44 setae. Chaetotaxy and measurements of tergites are given in Tables 5 and 6.

**Table 6.** Chaetotaxy of tergites (holotype in brackets).

**No. of tergites**	**lateromarginal**	**Inserted seta**	**Central setae**	**Other setae**
1^st^				
2^nd^	7–8 (8)	1–2 (2)	2 (2)	9–12 (9)
3^rd^	8–9 (9)	2 (2)	2–3 (3)	17–28 (17)
4^th^	6–7 (7)	1 (1)	3–4 (4)	11–16 (16)
5^th^	6–7 (7)	1–2 (2)	4–7 (7)	15–17 (17)
6^th^	8–11 (11)	2 (2)	3–5 (5)	26–36 (36)
7^th^	6–7 (6–7)	1–2 (2)	3–5 (5)	15–18 (18)
8^th^	6–8 (8)	1–2 (2)	4–5 (5)	13–15 (15)
9^th^	9–12 (11–12)	1–2 (2)	4 (4)	28–36 (36)
10^th^	6–7 (7)	1–2 (1–2)	4–5 (5)	14–17 (17)
11^th^	6–7 (7)	2 (2)	4 (4)	13–15 (15)
12^th^	8–10 (10)	1–2 (2)	4 (4)	22–33 (33)
13^th^	6–7 (6)	1–2 (2)	3–6 (6)	12–14 (14)
14^th^	13–18 (18)*			7–15 (15)
15^th^	7–9 (8–9)	1–2 (1)	2–4 (4)	18–26 (26)
16^th^	10–15 (15)*			6–8 (8)
17^th^				32–44 (44)

Note: * – marginal setae.

*Legs.* 1^st^ pair of legs reduced to two small hairy cupules, each with two long setae (Fig. 3G). Basal areas of legs 2–12 each with 5–7 setae. Leg 12 about 0.1 times the length of the body, about same length like head. Trochanter distinctly longer than wide (70–83 μm, 40–50 μm), with 7 or 8 subequal setae. Femur as long as wide (38–43 μm, 35–40 μm), with 5 setae and one of them (22 μm) distinctly longer than others (12–18 μm); trochanter and femur pubescent dorsally, ventrally with cuticular thickenings in pattern of large scales. Tibia nearly 2 times longer than wide (50–60 μm, 27–30 μm), with 6 dorsal setae and longest one nearly the same length as greatest diameter of tibia (21–30 μm). Tarsus subcylindrical, about 3.5 times as long as wide (68–75 μm, 20 μm) with 5 dorsal setae: 3 straight and protruding, 2 curved and depressed; longest setae (20–22 μm) about same length of greatest width of podomere; 2 ventral setae inserted near claw distinctly shorter than dorsal ones. Claws curved, anterior one somewhat longer and broader than posterior one, the latter more curved than the former (Fig. 4G). All legs covered with dense pubescences except areas with cuticular thickenings.

*Coxal sacs* present at bases of 3^rd^–9^th^ pairs of legs, fully developed, each with 4 setae on surface (Fig. 3G).

*Styli* present at base of 3^rd^–12^th^ pairs of legs, subconical (length 5–8 μm, width 3–4 μm), basal part with straight hairs; distal quarter hairless and blunt (3–4 μm) (Figs 3G, 4F).

*Sensecalicles* with smooth margin to pit, about same length as outer diameter (28–30 μm, 26–30 μm). Sensory seta inserted in cup center, extremely long, length 170–180 μm, at least 5.5 times longer than other two lateral setae (20–24 μm, 13–20 μm respectively) inserted at edge of cup.

*Cerci* subuliform, about 0.7 of head length and leg 12, length at least three times as long as its greatest width (150–188 μm, 45–58 μm), moderately covered with subequal length and slightly curved setae, with longest (25–28 μm) at least half of greatest width of cerci, terminal area (24–28 μm) short, circled by 6–8 layers of curved ridges. Terminal setae length 25 μm (Fig. 4H).

###### Etymology.

We dedicate this new species in honor of the late Professor Zhong Yang (1964–2017) who was an eminent botanist from Fudan University, for his great contribution to the knowledge of flora and biodiversity of Tibet.

###### Distribution.

Known only from the type locality.

###### Remarks.

*Symphylellazhongi* sp. n. is most similar to *S.multisetosa* Scheller, 1971 in the shape of the Tömösváry organ, as well as the shape and chaetotaxy of the tergites 1–4, 1^st^ leg, but it deviates distinctly in the shape of the ends of the processes (with ovoid swollen ends in *S.zhongi* sp. n. vs without ovoid swollen ends in *S.multisetosa*), chaetotaxy of the cerci (most setae subequal length and slightly curved in *S.zhongi* sp. n. vs long, straight, erect setae on dorsal, ventral, and outer sides of cerci arranged in one or two rows in *S.multisetosa*). It is also similar to *S.simplex* (Hansen, 1903) in the shape and chaetotaxy of first two tergites, sensory organs of antennal segments and 1^st^ leg, but differs in the shape of 1^st^antennal segment (moderate in *S.zhongi* sp. n. vs very short in *S.simplex*), chaetatoxy of 3^rd^ tergite (8 or 9 lateromarginal setae in *S.zhongi* sp. n. vs 11 or 12 in *S.simplex*) and 4^th^ tergite (6 or 7 lateromarginal setae in *S.zhongi* sp. n. vs 8 in *S.simplex*), chaetotaxy of cerci (8–10 longish setae protruding, others short and depressed in *S.simplex*).

The new species is compared with similar species in Table 7.

**Table 7.** Comparison of *S.macropora* sp. n., *S.zhongi* sp. n. and the similar species.

**Characters**	***S.macropora* sp. n.**	***S.javanens***is	*** S. asiatica ***	***S.zhongi* sp. n.**	*** S. multisetosa ***	*** S. simplex ***
Tömösváry organ	Oval, with large and elongated oval openings	Subspherical, openings middle size and flat	Openings small	Globular, openings small and roundish	Opening small	Opening moderate size
Central rod	Both anterior and posterior portions distinct	Anterior half and anterior branches very thin with traces	Both anterior and posterior portions distinct	Both anterior and posterior portions distinct	Both anterior and posterior portions distinct	Both anterior and posterior portions distinct
Processes on tergites 2–4	Broad	Broad	Broad	Slender	Slender	Slender
Stylus	Apex blunt	Apex truncate	Short	Apex blunt	Slender	?
Mushroom-shaped organs on antenna	Absent	?	?	Present	?	?
End of processes	Without swollen ends	With small glabrous triangular or ovoid swollen ends	with small swollen ends	With ovoid swollen ends	Without swollen ends	With small swollen ends
Setae of cerci	Subequal length and slightly curved	Mainly short, thin, slightly curved	Long and erect setae on dorsal, ventral and outer sides arranged in 1 or 2 rows	Subequal length and slightly curved	Long, straight , erect setae on dorsal, ventral and outer sides arranged in 1 or2 rows	8–10 longish setae protruding, others short and depressed

The 43 species of the genus *Symphylella* can be distinguished by the following key, but six species (*Symphylellanatala* Hilton, 1938, *Symphylellavaca* Hilton, 1938, *Symphylellaelongata* Scheller, 1952, *Symphylellafoucquei* Jupeau, 1954, *Symphylellamaorica* Adam & Burtel, 1956, and *Symphylella* sp. Rochaix, 1956), which have very brief original descriptions, are not included.

#### Key to the species of the genus *Symphylella*

**Table:** 

1	Without seta between inner basal setae and apical setae	**2**
–	At least with one seta between inner basal setae and apical setae	**3**
2	Setae on antennae plumose	***S.plumosa* Scheller, 1971**
–	Setae on antennae glabrous	**4**
3	Processes of anterior tergites blunt or broad	**10**
–	Processes of anterior tergites slender or prominent	**27**
4	Lateral margins of tergites slightly concave as in the genus *Scolopendrellopsis*	***S.tenuis* Scheller, 1961**
–	Lateral margins of tergites not concave	**5**
5	Cerci at least 3 times longer than wide	**6**
–	Length of cerci less than 3 times of width	**7**
6	Central rod with only hind part visible	***S.cylindrica* Scheller, 1961**
–	Central rod distinct	**8**
7	Anterior branch of central rod well developed, cerci with dense setae	***S.bornemisszai* Scheller, 1961**
–	Anterior branch of central rod indistinct, cerci with sparse setae	**9**
8	Cerci with strongly bulging outer sides	***S.abbreviata* Scheller, 1971**
–	Cerci without strongly bulging outer sides	***S.hintoni* Edwards, 1958**
9	Central rod interrupted in the middle	***S.australiensis* Scheller, 1961**
–	Central rod complete	***S.oligosetosa* Scheller, 1971**
10	Several setae on antennae with fine hairs	***S.antennata* (Hansen, 1903)**
–	All setae on antennae normal	**11**
11	Long lateral setae present between tergites 2–3, 3–4, 6–7	***S.santa* Hilton, 1931**
–	Long lateral setae absent between tergites 2–3, 3–4, 6–7	**12**
12	Central rod with three branches caudally	***S.cubae* Hilton, 1931**
–	Central rod not branched caudally	**13**
13	Anterior branch of central rod faint, indistinct, or only trace visible	**14**
–	Anterior branch of central rod distinct or well developed	**18**
14	Cerci with dense setae, setae between inner basal setae and apical setae as long as anterior lateral setae	***S.erecta* Domínguez Camacho, 2012**
–	Cerci with moderate setae, setae between inner basal setae and apical setae distinctly shorter than anterior lateral setae	**15**
15	Processes without swollen ends; inner margins of openings on Tömösváry organs covered by minute irregular teeth	***S.macropora* sp. n.**
–	Processes with swollen ends, inner margins of openings on Tömösváry organs without teeth	**16**
16	First tergite with 5 setae	***S.lubumbashi* Domínguez Camacho, 2012**
–	First tergite with at least 6 setae	**17**
17	Apex of styli spatulate	***S.javanensis* Scheller, 1988**
–	Apex of styli pointed	***S.asiatica* Scheller, 1971**
18	Length of cerci less than 3 times of greatest width	**19**
–	Cerci at least 3 times longer than greatest width	**21**
19	Central rod interrupted medially, anterior lines extending laterally to near insertion of antennae, lines also extending laterally from the mid-point interruption of the central rod and then diagonally to near insertion of antennae	***S.delawarensis* Allen & Walther, 1993**
–	Central rod interrupted medially, without any lateral line through it	**20**
20	Cerci with dense setae, slightly curved and depressed on all sides, one erect and longer seta on ventral side	***S.fuko* Domínguez Camacho, 2012**
–	Cerci with moderate setae, outer side with one protruding setae in the distal part, ventral with five setae arranged in a longitudinal row	***S.tanganyika* Domínguez Camacho, 2012**
21	Cerci 4 times longer than greatest width, with densely setae	**22**
–	Length of cerci less than 4 times of greatest width, with moderate or sparse setae	**23**
22	Anterior lateral setae short, less than 0.5 of processes	***S.texana* (Hansen, 1903)**
–	Anterior lateral setae long, at least 0.5 of processes	***S.isabellae* (Grassi, 1886)**
23	Cerci with all setae slightly curved and depressed, central rod distinct posteriorly only	***S.malagassa* Domínguez Camacho, 2012**
–	Cerci with two kinds of setae, slightly curved setae and erect long setae	**24**
24	Processes without swollen ends	***S.tenella* Scheller, 1961**
–	Processes with swollen ends	**25**
25	Cerci with sparse setae	***S.kalundu* Domínguez Camacho, 2012**
–	Cerci with moderate setae	**26**
26	With 1–2 setae between inner basal setae and apical setae	***S.vulgaris* (Hansen, 1903)**
–	With at least 2 setae between inner basal setae and apical setae	***S.neotropica* (Hansen, 1903)**
27	Setae on tergites very long	***S.longiseta* Michelbacher, 1941**
–	Setae on tergites in moderate length	**28**
28	Fine sutures or lines that apparently passes through the interruption and continues on either side perpendicularly to the central rod for some distance and then turns obliquely forward ending at the side of the Tömösváry organs	***S.sierra* Michelbacher, 1939**
–	Without any sutures or lines connected with the central rod	**29**
29	Central rod not or hardly divided in the middle	**30**
–	Central rod divided distinctly in the middle	**31**
30	Tergite 2 with only 5 lateral setae	***S.capicola* Michelbacher, 1942**
–	Tergite 2 with 7-8 lateral setae	***S.rossi* Michelbacher, 1942**
31	Each tergites at least has 2 setae between inner basal setae and apical setae	**32**
–	1-3 setae between inner basal setae and apical setae	**33**
32	Anterior lateral setae much shorter than processes	***S.subterranean* Michelbacher, 1939**
–	Anterior lateral setae at least 2/3 length of processes	***S.oviceps* Michelbacher, 1939**
33	Lateral margin of cerci nearly straight	***S.major* Scheller, 1961**
–	Lateral margin of cerci slightly curved	**34**
34	Anterior branch of central rod vestigial	**35**
–	Anterior branch of central rod well developed	**36**
35	Posterior part of central rod very thick, dorsal side of head with sparse setae	***S.tentabundna* Scheller, 1971**
–	Posterior part of central rod normal, dorsal side of head with dense setae	***S.multisetosa* Scheller, 1971**
36	Anterior branch of central rod faint	**41**
–	Anterior branch of central rod distinct	**37**
37	Tergite 4 with 5 lateral setae	**38**
–	Tergite 4 with at least 6 lateral setae	**42**
38	Cerci with sparse setae	***S.geum* Michelbacher, 1941**
–	Cerci with moderate or dense setae	**39**
39	Anterior part of central rod slender	***S.brevipes* (Hansen, 1903)**
–	Anterior part of central rod normal	**40**
40	Length of cerci more than 3.5 times of greatest width	***S.pusilla* (Hansen, 1903)**
–	Length of cerci less than 3.5 times of greatest width	***S.brincki* Scheller, 1971**
41	Body length 4–4.8 mm; antennae setae on inner side of basal segments about 1.5 times as long as those on outer side	***S.essigi* Michelbacher, 1939**
–	Body length 3 mm; antennae setae on inner side of basal segments slightly longer than those on outer side.	***S.capitata* Michelbacher, 1939**
42	Anterior branch of central rod thin, cerci at least 4 times longer than greatest of width	***S.simplex* (Hansen, 1903)**
–	Anterior branch of central rod well developed, cerci less than 4 times of greatest width	***S.zhongi* sp. n.**

## Supplementary Material

XML Treatment for Symphylella

XML Treatment for Symphylella macropora

XML Treatment for Symphylella zhongi
